# Influence of inoculation with *Lactobacillus* on fermentation, production of 1,2-propanediol and 1-propanol as well as Maize silage aerobic stability

**DOI:** 10.1515/biol-2020-0038

**Published:** 2020-06-11

**Authors:** Marek Selwet

**Affiliations:** Department of General and Environmental Microbiology, Poznań University of Life Sciences, ul. Szydłowska 28, 60-637 Poznań, Poland

**Keywords:** *Lactobacillus buchneri*, aerobic stability, silage

## Abstract

The aim of this study is to determine the influence of a commercial bacterial inoculant (L1) and a preparation (L2) containing three *Lactobacillus* strains capable of producing 1,2-propanediol and short-chain fatty acids on maize silage aerobic stability improvement. The research showed that during 90-day ensilage, the applied preparations significantly reduced the content of DM, water-soluble carbohydrates (WSCs), pH and DM recovery (*P* < 0.05). The concentration of lactic acid (LA), acetic acid (AA) and propionic acid (PA) in the inoculated samples increased significantly (*P* < 0.05). 1,2-Propandiol and 1-propanol were not found in control silages (without additives). The addition of L1 and L2 significantly (*P* < 0.05) increased the concentration of these substances. The L1 and L2 mixtures significantly extended (*P* < 0.05) the silage aerobic stability.

## 1 Introduction

Lactic acid-fermenting bacteria are used as a major microbiological modifier that could improve the silage chemical composition [1]. These bacteria may increase the dry matter recovery rate [2] and improve the hygienic state of silage, which is determined by the content of moulds and yeasts [3]. It is noteworthy that acetic acid produced by heterofermentative lactic acid bacteria may alter the yeast metabolism and improve the silage aerobic stability. However, heterofermentative bacterial strains do not metabolise lactic acid efficiently as they consume large amount of energy in this process. It is a disadvantage, which causes a greater loss of nutrients [4]. Therefore, it seems reasonable to use adequate mixtures of lactic heterofermentative and homofermentative strains depending on the ensiled plant material [5]. Alternatively, enzyme preparations can be used, but they are more expensive and it is more difficult to prepare them [6]. There is a considerable divergence in the results of the latest research concerning the type of additives used. They are divided into different groups: ‘homolactic’ (homolactic bacteria), ‘hetero’ (heterofermentative bacteria), ‘combo’ (homolactic plus heterofermentative bacteria) and ‘chemical’ (chemical additives) [7]. Homofermentative bacterial inoculants ferment water-soluble carbohydrates into organic acids, especially lactic acid, which quickly acidifies silage and inhibits the undesirable bacteria growth. Heterolactic bacterial inoculants ferment water-soluble carbohydrates into antifungal acids, such as acetic and propionic acids, which inhibit the growth of spoilage-causing fungi. Commercially available inoculants contain one or both types of lactic acid bacteria. So far, few studies have simultaneously compared several commercially available inoculants with chemical additives [8]. Recently, there have been numerous studies on the use of the selected lactic acid bacterial strains, mainly *Lactobacillus buchneri*. According to the results, this heterofermentative strain improves the silage aerobic stability. Apart from that, it anaerobically degrades lactic acid to acetic acid and 1,2-propanediol. Probably, 1,2-propanediol is an intermediate metabolite, which becomes degraded into 1-propanol and propionic acid by *Lactobacillus diolivorans* [9]. *Lactobacillus reuteri* can synthesise cobalamin, which is a coenzyme for diol dehydratase – the enzyme catalysing 1,2-propanediol conversion into 1-propanol and propionic acid [10]. During co-fermentation, the synthesis of acetic acid, 1,2-propanediol and propionic acid is stimulated by bacterial strains from these species. They improve the aerobic stability of renewable feed silage. The research results were used to make bacterial preparations stimulating the ensilage of renewable raw materials [11].

The aim of this study is to determine the influence of a commercially available bacterial preparation and a mixture of *L. buchneri* strains on 1,2-propanediol and 1-propanol, the chemical composition and aerobic stability of maize silages.

## 2 Materials and methods

### 2.1 Plant material

Maize (*Zea mays* L.) SAN cultivar (FAO 240) from the Hodowla Roślin in Smolice Ltd/Sp. z o.o. IHAR Group was ensilage. Type of use: medium-early three-line hybrid (TC) with advantages of a grain hybrid for CCM and silage. The features of maize were as follows: high yields of total dry matter and dry matter of cobs, high resistance to *Fusarium* stem rot and root lodging, tolerant to smut, long-lasting green leaves and stems and the height of 270 cm. Plant density is 150,000 per ha. Maize was harvested in October at the end of silage maturity (BBCH 83). It was cut/harvested at a height of 40 cm. Before ensiling, it was cut into 2–3 cm chaff. Maize was grown in monoculture.

### 2.2 Bacterial preparations used to silage inoculation

L1 is a commercially available preparation containing in lyophilisate the following cultures: *Lactobacillus plantarum* K KKP/593/p, *L. plantarum* C KKP/788/p, *Lactobacillus brevis* KKP 839 and *L. buchneri* KKP 907. The producer recommended a dose of 5 g t^−1^ of ensiled material. The concentration of bacteria in 1 g of the preparation was 10^9^ cfu g^−1^.

L2 is a mixture (lyophilisate) of three strains: *L. buchneri* ATCC 4005, *Lactobacillus dioliovorans* LGM 19667, and *L. reuteri* ATCC 23272 (DSMZ). A dose of 5 g t^−1^ of ensiled material was used. The concentration of bacteria in 1 g of the mixture was 10^9^ cfu g^−1^.

### 2.3 Ensilage and determination of aerobic stability

Silages were prepared in PVC laboratory micro silos with a capacity of 4 dm^3^ equipped in a closure allowing removal of gaseous products. The average temperature during ensiling was 20 ± 1°C. During the aerobic stability test, samples were aerated for 7 days at 20°C. After this period, changes in microorganism counts and silage selected chemical parameters were investigated. After 90-day ensilage, moist samples weighing 85 g were removed from micro silos and placed in 500 mL plastic containers with 4 mm holes enabling air circulation. The temperature was measured with a temperature reader (Hotmux DDC Corporation, Pennsauken, NJ, USA) every 5 min at 2 h intervals. Stability was defined as the time necessary to raise silage temperature by ≥2°C relative to the ambient temperature. The number of replications was 5.

### 2.4 Microbiological and chemical analyses

Lactic fermentation bacteria were cultured on MRS Agar (Oxoid). Incubation time was 48 h at 35°C. Yeasts and moulds were cultured on OGYE Agar (Oxoid) with oxytetracycline (oxytetracycline-glucose-yeast-extract agar). Incubation time was 5 days at 25°C.

Lactic acid, acetic acid, propionic acid, ethanol, 1-propanol and 1,2-propanediol concentrations were measured with a gas chromatograph equipped with an FID detector, 2 m long 80/100 Chromosorb® WAW glass column (Supelco), I.D. 2 mm with GP 10% SP–1,200/1% H_3_PO_4_ filling and Varian 8200 CX autosampler. Hydrogen was used as the carrier gas (flow 30 cm^3^ min^−1^) with oven temperature of 120°C, injection temperature of 250°C and detector temperature of 300°C. Fluka acid standards were used.

The basic composition of feeds was determined in accordance with AOAC [12]. pH values were measured with a pH meter (Hann Instruments) in a suspension prepared from 20 g of silage and 180 cm^3^ of demineralised water, which was homogenised for 10 min.

### 2.5 Statistical analysis

The GLM SAS procedure package was used for statistical calculations [13]. Differences between the means were tested using Tukey’s test.

## 3 Results

Basic chemical composition and counts of lactic acid bacteria, yeast and mould in the ensilaged maize forage are presented in Table 1.

**Table 1:** The chemical composition and count of microorganisms in maize green forage before ensilage

Whole crop maize	
DM (g kg^−1^)	404
pH	5.62
CP (g kg^−1^)	93
WSC (g kg^−1^)	74.6
LAB log CFU g^−1^	6.12
Yeast log CFU g^−1^	7.2
Mould log CFU g^−1^	6.1

DM = dry matter, CP = crude protein, WSC = water-soluble carbohydrates, LAB = lactic acid bacteria.

Table 2 presents the chemical composition and counts of microorganisms after 90-day ensilage. Dry matter concentration and WSC in silages treated with bacterial inoculants L1 and L2 were significantly lower (*P* < 0.05) than in the control sample. The silages with *Lactobacillus* strains had significantly (*P* < 0.05) lower pH values and a lower DM recovery index. Also, concentrations of LA, AA and PA were significantly (*P* < 0.05) higher in the inoculated samples. The inoculation did not have a significant effect (*P* > 0.05) on the content of ethanol and CP in silages. Mixtures of *Lactobacillus* strains caused a significant (*P* < 0.05) increase in the LAB population and a decrease (*P* < 0.05) in the population of yeasts and moulds. There was no 1,2-propandiol or 1-propanol found in control silage samples. The addition of L1 and L2 significantly (*P* < 0.05) increased concentrations of these substances. The content of 1,2-propandiol and 1-propanol in the combination with L2 was relatively 62% and 75%, respectively, greater than in the combination with L1.

**Table 2:** The effect of inoculation with different *Lactobacillus* strains on the quality, chemical composition and count of microorganisms in maize silage

Parameters	Treatments		
	Control	L1	L2
DM (g kg^−1^)	385^a^	370^b^	372^b^
pH	4.15^a^	3.97^b^	3.82^b^
CP (g kg^−1^ DM)	92.8^a^	93^a^	92.7^a^
WSC (g kg^−1^ DM)	42.7^a^	36.8^b^	32.3^b^
LA % DM	5.2^b^	6.8^a^	6.9^a^
AA % DM	1^b^	2.7^a^	3.1^a^
PA % DM	0^c^	1.0^b^	1.4^a^
1,2-Propandiol % DM	0^c^	0.51^b^	1.5^a^
1-Propanol % DM	0^c^	0.2^b^	0.8^a^
Ethanol % DM	0.9^a^	0.7^a^	0.8^a^
DM recovery (g kg^−1^ DM)	95.29^a^	91.58^b^	92.08^b^
LAB log CFU g^−1^	6.55^a^	8.35^b^	8.42^b^
Yeast log CFU g^−1^	5.11^a^	4.25^b^	3.97^b^
Mould log CFU g^−1^	5.12^a^	5^a^	4.7^b^

LA = lactic acid, AA = acetic acid, PA = propionic acid, LAB = lactic acid bacteria.

^a,b,c^ Means marked with different letters in a row are different at *P* < 0.05.

Table 3 lists changes in silages exposed to oxygen. Inoculation with L1 and L2 strains significantly (*P* < 0.05) reduced the growth of pH in silages. AA and PA concentrations in the samples with *Lactobacillus* strains were significantly (*P* < 0.05) greater than in the control sample, but the LA content was significantly (*P* < 0.05) reduced. LAB counts in the inoculated silages were significantly (*P* < 0.05) greater, whereas yeast and mould counts were lower (*P* < 0.05) than in the control samples. No 1,2-propandiol or 1-propanol was found in the control samples. The content of 1,2-propandiol and 1-propanol in the combinations with L1 and L2 was the same as in silages before the aerobic stability test.

**Table 3:** The chemical composition and count of microorganisms in maize silage after the aerobic stability test

Parameters	Treatments		
	Control	L1	L2
pH	6.2^a^	5.01^b^	4.99^b^
LA % DM	5.1^a^	5.8^a^	4.6^b^
AA % DM	1.7^c^	3.5^b^	4.9^a^
PA % DM	0^c^	0.8^b^	1.0^a^
1,2-Propandiol % DM	0^c^	0.5^b^	1.5^a^
1-Propanol % DM	0^c^	0.2^b^	0.8^a^
LAB log CFU g^−1^	6.01^b^	8.02^a^	8.1^a,b^
Yeast log CFU g^−1^	8.4^a^	7.13^b^	6.15^c^
Mould log CFU g^−1^	7.17^a^	6.18^b^	5.02^c^

LA = lactic acid, AA = acetic acid, PA = propionic acid, LAB = lactic acid bacteria.

^a,b c^ Means marked with different letters in a row are different at *P* < 0.05.

**Figure 1:**
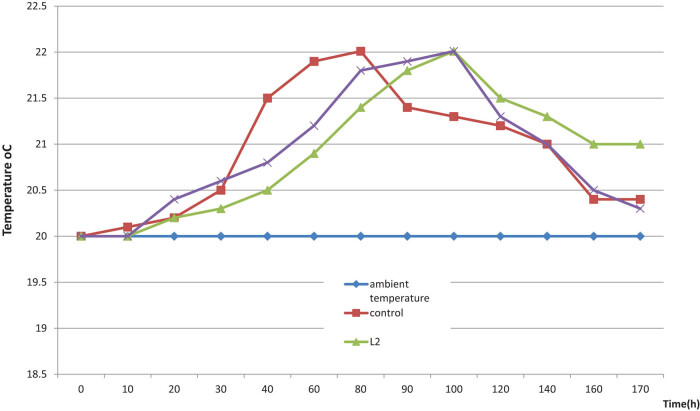
Variation in the temperature of maize silages during the aerobic stability test.

Figure 1 shows temperatures taken during measurements of the silage aerobic stability. L1 and L2 mixtures significantly (*P* < 0.05) extended the aerobic stability. The silage temperature in control samples increased by 2°C within 72 h. The inoculated silages were characterised by longer stability, i.e., 103 h for L1 and 102 h for L2.

## 4 Discussion

According to the study by Rezende et al. [14], when silages are exposed to air, considerable changes in their chemical composition (significant increase in pH) occur and their temperature increases considerably during exposure to oxygen.

Many strains of *Lactobacillus* can be used to improve the silage aerobic stability of maize. In our experiment, the effect of a commercially available preparation was compared with a preparation containing heterofermentative strains, namely, *L. buchneri*, *L. dioliovorans* and *L. reuteri*. Zielińska et al. [15] and Muck et al. [16] described the synergistic effects of the combination of various *Lactobacillus* strains and their improvement of silage stability. It seems very important that these strains can metabolise 1,2-propandiol into propionic acid and 1-propanol. According to the scientific reports, *L. buchneri* [17], *L. dioliovorans* [18] and *L. reuteri* [10,19,20] exhibit these properties.

Driehuis et al. [21] and Jungbluth et al. [22] observed that *L. buchneri* strains increased acetic acid and 1,2-propandiol concentrations and decreased lactic acid content in silages. Our research findings were similar. However, it is noteworthy that too high concentrations of acetic acid may affect the silage taste.

Oliveira et al. [23] observed that when one strain or a mixture of *Lactobacillus* strains were applied to silages, they reduced pH values and WSC concentrations. The same observations were made in our research. However, contrary to the results of our research, Oliveira et al. also found a lower concentration of acetic acid in samples with *Lactobacillus* strains. Similar results were recorded when the concentration of lactic acid in the inoculated samples was greater than in the control samples. At the same time, these authors concluded that the observed effects depended on the type of plant ensiled.

When the aerobic stability of silages was checked, the concentration of acetic acid was found increased in inoculated samples (concentration of bacteria in 1 g of preparation was 10^9^ cfu g^−1^). Basso et al. [24] noted similar results but at a concentration of 5 × 10^5^ cfu g^−1^. According to the study by Ranjit and Kung [25], acetate production by *Lactobacillus* can be continued during exposure to oxygen. Acetic acid concentration tended to increase, whereas the content of lactic acid was found to decrease in inoculated silage samples subjected to aerobic incubation. In consequence, pH decreased because acetic acid exhibited higher p*K*_a_ values than lactic acid [9]. Inoculants used in our research did not affect changes in the CP content. Silva et al. [26] used *L. buchneri* strains and noted an increase in the CP level compared with that in the control sample. Similar results were reported by Bumbieris et al. [27], who observed that the CP content in inoculated samples (7.47%) was greater than in the control samples (6.87%). *Lactobacillus* strains used in our study reduced yeast and mould counts. The L2 preparation exhibited a stronger fungistatic effect. It is noteworthy that the production of substances inhibiting fungal growth, including acetic and propionic acid, may largely depend on the phase of their growth as well as temperature, chemical composition and pH of the substrate [28].

## 5 Conclusions

The performed research showed that silages inoculated with *Lactobacillus* strains revealed better aerobic stability than control samples due to higher acetic and propionic acid concentrations, which reduced pH as well as yeast and mould counts. At the same time, silages inoculated with heterofermentative strains of *Lactobacillus* had a higher content of 1,2-propandiol and 1-propanol. This fact may indicate that mixtures of these bacterial strains are excellent inoculants that improve the aerobic stability of silage.
